# Gene expression analysis of human adipose tissue-derived stem cells during the initial steps of *in vitro* osteogenesis

**DOI:** 10.1038/s41598-018-22991-6

**Published:** 2018-03-16

**Authors:** Anny Waloski Robert, Addeli Bez Batti Angulski, Lucia Spangenberg, Patrícia Shigunov, Isabela Tiemy Pereira, Paulo Sergio Loiacono Bettes, Hugo Naya, Alejandro Correa, Bruno Dallagiovanna, Marco Augusto Stimamiglio

**Affiliations:** 1 Instituto Carlos Chagas, Fiocruz-Paraná. Rua Professor Algacyr Munhoz Mader, 3775, Curitiba, PR 81350-010 Brazil; 2 Unidad de Bioinformática, Institut Pasteur Montevideo. Mataojo 2020, Montevideo, 11400 Uruguay; 3 Cirurgia Plástica Dr. Paulo Bettes, Rua Francisco Rocha, 1312, Curitiba, PR 80730-390 Brazil

## Abstract

Mesenchymal stem cells (MSCs) have been widely studied with regard to their potential use in cell therapy protocols and regenerative medicine. However, a better comprehension about the factors and molecular mechanisms driving cell differentiation is now mandatory to improve our chance to manipulate MSC behavior and to benefit future applications. In this work, we aimed to study gene regulatory networks at an early step of osteogenic differentiation. Therefore, we analyzed both the total mRNA and the mRNA fraction associated with polysomes on human adipose tissue-derived stem cells (hASCs) at 24 h of osteogenesis induction. The RNA-seq results evidenced that hASC fate is not compromised with osteogenesis at this time and that 21 days of continuous cell culture stimuli are necessary for full osteogenic differentiation of hASCs. Furthermore, early stages of osteogenesis induction involved gene regulation that was linked to the management of cell behavior in culture, such as the control of cell adhesion and proliferation. In conclusion, although discrete initial gene regulation related to osteogenesis occur, the first 24 h of induction is not sufficient to trigger and drive *in vitro* osteogenic differentiation of hASCs.

## Introduction

Mesenchymal stem cells (MSCs), including human adipose tissue-derived stem cells (hASCs), are undifferentiated cell populations characterized by the ability to undergo self-renewal and the capacity for multilineage differentiation^1^. hASCs can be found in adipose tissue in large amounts (millions to billions of cells), and they can be collected and harvested by a minimally invasive procedure. Due to their multipotent nature, when appropriately stimulated *in vitro*, they can differentiate into several tissue-specific lineages, including the chondrogenic, osteogenic and adipogenic lineages^2^. All these characteristics make these cells very valuable for cell-based medical therapies. Among clinical applications, the use of MSCs for bone regeneration has been gaining attention^3^. Understanding the biological pathways and transcription factors involved in determining the osteogenic fate is key to future advances and improvements of bone regeneration therapies.

Currently, there are many questions remaining about how to control the stem cell switch from self-renewal to differentiation *in vitro*. The fate and function of an *in vitro* MSC culture depend on many external factors, including the cell culture media and supplements (i.e., growth factors), mechano-electro stimuli, and the use of three-dimensional scaffolds^4^. Moreover, little is known about how cell fate determinants are regulated in functionally important gene regulatory networks, which remains a challenge. Gene expression analysis, both genome-wide and targeted at specific gene subsets, has played a key role in improving our understanding into the molecular pathways involved in hASCs self-renewal and differentiation^5^.

Considering the osteogenic differentiation of MSCs, there are a plethora of signals and molecular pathways described in the literature. Signaling pathways including TGFβ/BMP signaling, Wnt signaling, Hedgehogs, Notch, and FGFs have been involved (reviewed by Wu and colleagues, 2016^6^). Recently, Yong and coworkers (2016) demonstrated the influence of ERK1/2 and PKA signaling pathways to the induction of osteogenesis on murine ASCs under an electromagnetic field (EMF) stimulus^7^. Accordingly, Fathi and Farahzadi (2017) confirmed Yong’s findings demonstrating that zinc sulphate, in the presence of EMF, induce the expression of osteogenic genes on murine ASCs via PKA, ERK1/2 and Wnt/β-catenin signaling pathways^8^.

The study of MSC differentiation and maturation has also been globally monitored by gene expression profiles that have historically focused on measuring total mRNA levels through high-throughput analyses. Using microarray expression profiling, van de Peppel and colleagues (2017) identified gene regulatory events during osteogenic and adipogenic lineage commitment of MSCs. Their data analysis revealed that expression levels of identified transcription factors did not always change and indicate additional post-transcriptional regulatory mechanisms during differentiation^9^. In fact, recent studies reveal that steady-state mRNA levels only loosely correspond to the composition of the proteome^10,11^, thereby indicating that post-transcriptional mechanisms play a major role in the regulation of gene expression. Until recently, precisely monitoring translation was far more challenging than measuring mRNA levels. However, this scenario has changed with the development of the polysome profiling approach^12^. The analysis of the mRNA fraction associated with polysomes has been used as a strategy to analyze posttranscriptional mechanisms involved in translational control as well as to quantitatively measure expression^13^. We previously demonstrated that, in a model of adipogenic differentiation, RNA-seq analysis of the mRNA fraction associated with polysomes showed a significant percentage of regulated mRNAs that were controlled at the translational level and/or by changes in their transcript levels^12^. Additionally, this previous work demonstrated that three days of *in vitro* cell differentiation induction with adipogenic stimulation medium are sufficient for the initiation of adipogenesis and the upregulation of its correlated gene networks.

In this work, we aimed to investigate genes and pathways involved in driving the initial trigger of osteogenic differentiation of hASCs. For this purpose, we focused on the early stages of osteogenic differentiation and analyzed both the total mRNA and the mRNA fraction associated with polysomes. Our question was whether gene network regulation as early as 24 h after induction could determine hASCs commitment to *in vitro* osteogenesis. Moreover, we aimed to assess how relevant the first differentiation signals could be to modulate hASC behavior in culture during osteogenesis induction.

## Results and Discussion

### Early stages of osteogenic differentiation display slight differences in gene regulation

The identity of the hASCs used in this work was confirmed by the expression of cell-surface antigens and by their capacity to undergo adipogenic and osteogenic differentiation. The immunophenotype profiles of the cells indicated that hASCs were uniformly positive for the membrane glycoprotein CD90, the endoglin receptor CD105 and the surface enzyme ecto-5′-nucleotidase CD73, confirming their canonical MSC characteristics (see Supplementary Fig. S1A). No detectable contamination by endothelial or hematopoietic cells was observed, as flow cytometry analysis was negative for CD31, CD19, CD34, CD117, CD11b, HLA-DR and CD45 markers (Supplementary Fig. S1A). The adipogenic and osteogenic differentiation potentials were verified by the presence of lipid-rich vacuoles stained with AdipoRed™ and by the presence of mineralized extracellular matrix stained with OsteoImage™ Mineralization, respectively (Supplementary Fig. S1B,C).

To understand the hASCs’ osteogenic differentiation, gene expression patterns were determined by studying the mRNA population associated with the translation machinery. In this work, we focused on the gene expression profile involved in the initial steps of osteogenesis. For this purpose, we allowed hASCs to differentiate *in vitro* in the presence of osteogenic induction media for 24 h. After this period of *in vitro* differentiation, it was not possible to observe any morphological differences or accumulation of mineralized extracellular matrix between the induced (IN) and non-induced cells (CT) (data not shown).

After 24 h of osteogenic induction, cells were subjected to isolation of total and polysomal RNA fractions by ultracentrifugation of cytoplasmic extracts into sucrose density gradients (10% to 50%). As previously demonstrated by our group^12^ and seen in this work, the hASCs had a polysome profile characteristic of cells with reduced levels of translational activity and with low amount of polysome complexes observed along the sucrose gradient (see Supplementary Fig. S2A).

Total and polysome-associated mRNA fractions were isolated and submitted to RNA-seq in an Illumina HiSeq platform. The total number of reads obtained for each sample is shown in Supplementary Table S1. The reads of all samples were mapped onto the reference genome (GRCh38), yielding a mean mapping percentage of approximately 80%, with approximately 17,000 genes detected.

**Figure 1:**
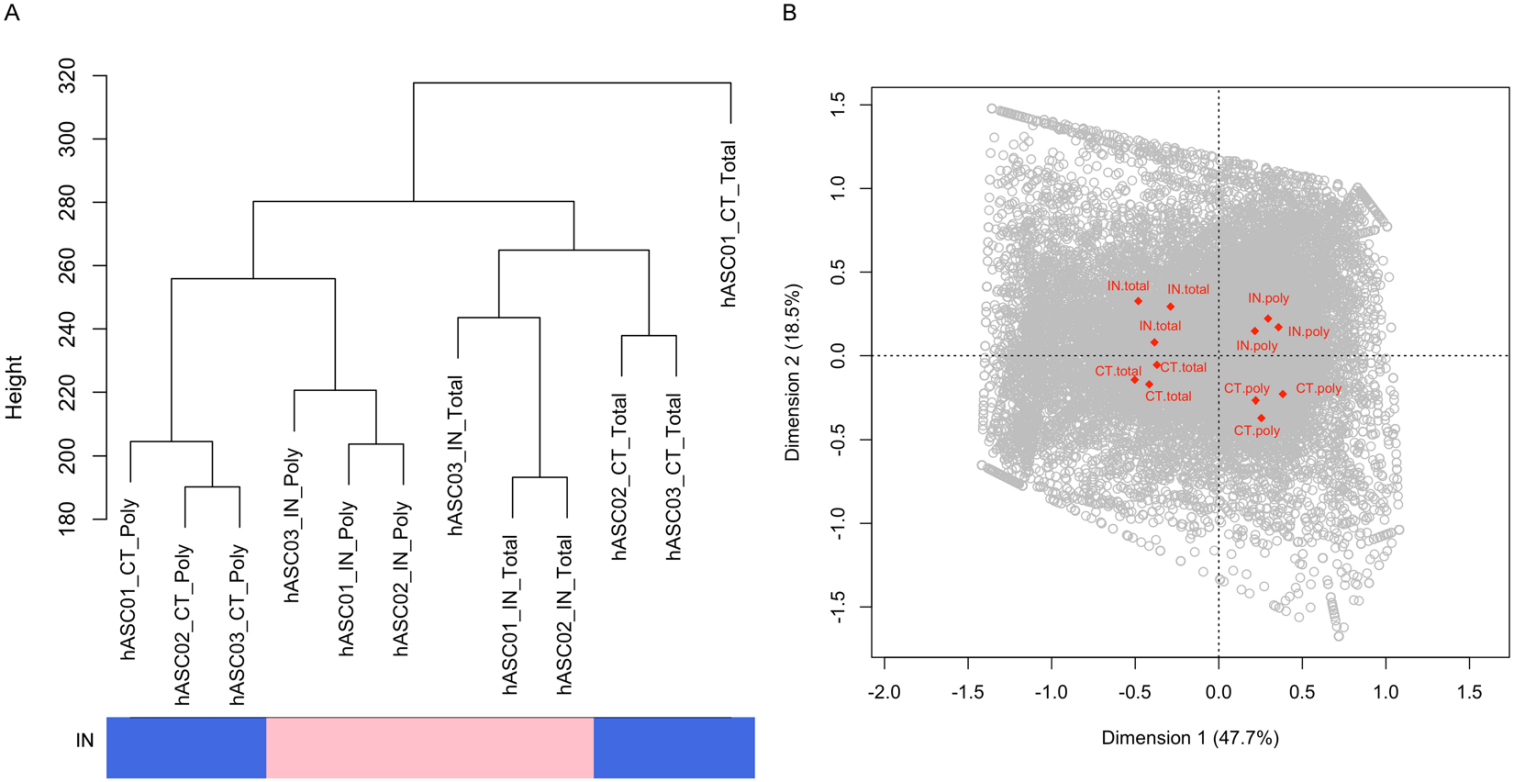
Results of hierarchical clustering and correspondence analysis showing the internal consistency of the data. (**A**) Dendrogram including all samples: CT and IN in the polysomal fraction and in total RNA (CT_Poly, IN_Poly and CT_Total, IN_Total, respectively). Log2 values of RPKM counts were used for the hierarchical clustering. The first branching event separates the fraction (polysomal from total) and the internal branching event separates the condition (CT and IN). This applies to all samples excluding TL01_CT_Total. (**B**) Correspondence analysis (COA) of samples. The first dimension (represented in the x-axis) separates RNA fraction (total vs. polysomal) and the second dimension (represented in the y-axis) separates the culture condition (CT vs IN).

Analyzing the total number of transcripts, we observed that most of the transcripts (14,711; ~95%) were detected in both samples (polysomal and total RNA fractions). Despite this, the expression levels were variable and some mRNAs were identified in only one RNA fraction (Supplementary Fig. S2B). Hierarchical clustering and correspondence analysis (COA) showed that samples were grouped first according to RNA fraction (total vs. polysomal) and then according to condition (CT vs. IN), indicating that, in all conditions, samples grouped according to the RNA fraction regardless of donor origin (Fig. 1A,B). Supporting our previous report^12^, these results also evidenced that total and polysomal RNA populations are intrinsically more characteristic than donor “idiosyncrasy”.

**Figure 2:**
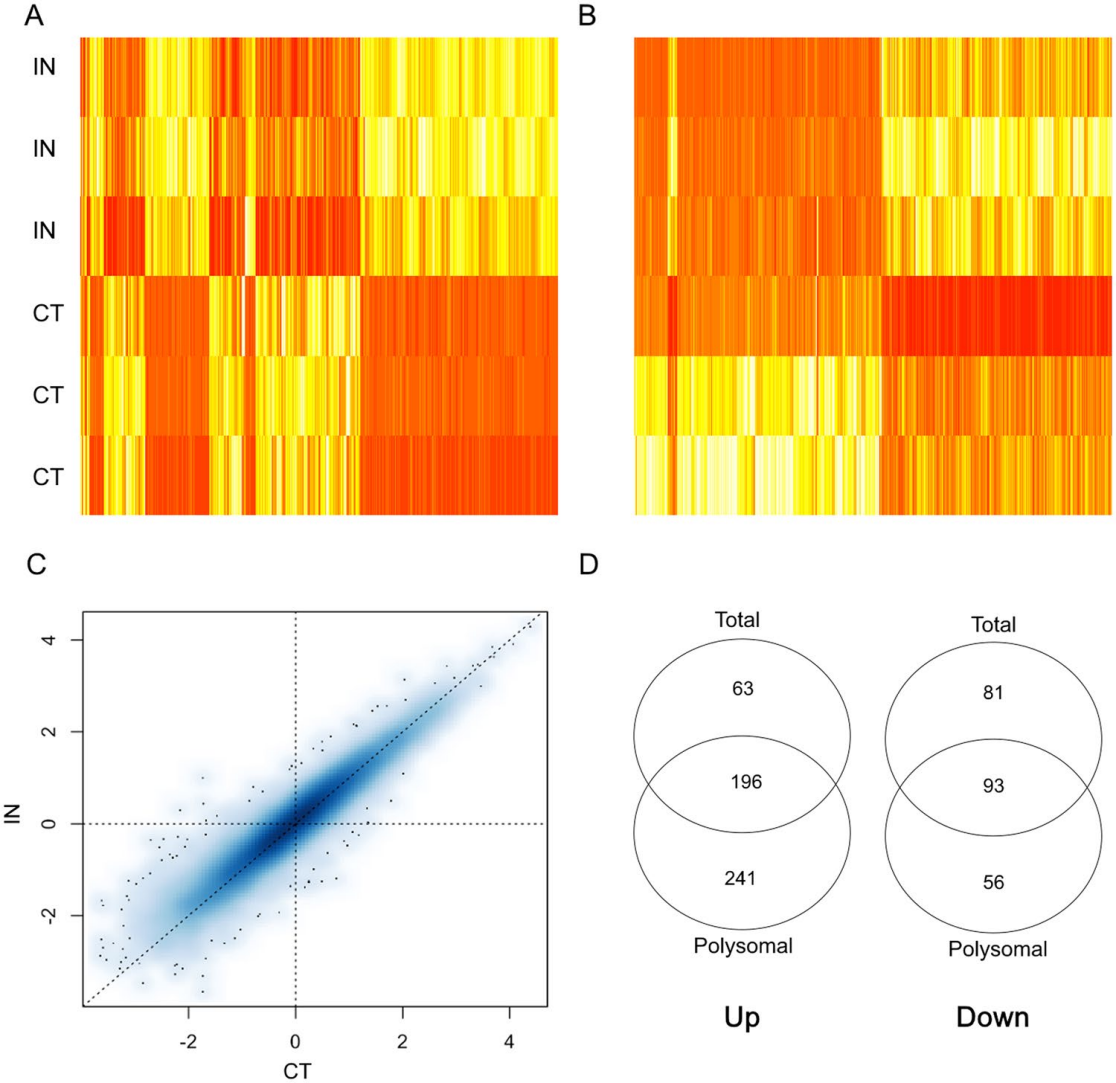
Diagrams for differentially expressed genes (DEGs) on osteogenic-induced (IN) and non-induced (CT) cells. Heatmaps of the DEGs are shown for the polysomal RNA fraction (**A**) and for the total RNA fraction (**B**). Data are displayed with different samples in the rows (three biological replicates for IN and for CT) and color intensities represent gene upregulation (red) and downregulation (yellow). (**C**) Scatterplot of log-10 RPKM values representing each gene in the polysomal fraction (median of reads per kilobase per million mapped reads from all patients for CT and IN samples). (**D**) Venn diagrams picturing set relationships between DEGs in the polysomal and total RNA fractions for upregulated genes (Up; left) and for downregulated genes (Down; right).

Differentially expressed genes (DEGs), considering an overall FDR ≤ 0.05 and a logFC ≥ ±2, were identified by paired comparisons between CT and IN conditions, and for polysomal and total RNA fractions (Supplementary Tables 2 and 3). Considering these results, representative heatmaps for total and polysomal RNA fractions allowed us to visualize the expression pattern of DEGs (Fig. 2A,B). Similar to previously reported^12^ and as expected, some differences in gene expression profile between induced and non-induced cells were noticed in both RNA fractions, but not between replicates. Also, RPKM values (log-10 reads per kilobase per million mapped reads) for polysomal RNA fraction in IN and CT conditions were illustrated in a scatter plot (Fig. 2C) showing that the expression levels of majority of genes are similar between conditions (blue central blur) and only some genes are differentially expressed (black points). Considering all analysis, we identified 586 DEGs in polysomal RNA fraction and 433 DEGs in total RNA fraction. Interestingly, comparing DEGs in total and polysomal RNA fractions, we observed differences in the quantity of up- and downregulated genes (Fig. 2D), with more upregulated subjects. In regard of this, Bionaz and coworkers (2015), evaluating differentiation of porcine ASCs, have described that osteogenesis is featured by a larger number of upregulated DEGs than downregulated ones at day 2 of *in vitro* differentiation. These data are compatible to that found in this work, but different from adipogenesis that display a large number of downregulated DEGs^14^.

Taken together, these RNA-seq results indicated that only 3–4% of the detected transcripts showed some degree of regulation in the first 24 h of differentiation. Our results suggest that most of this gene regulation is exerted through the control of transcript association with the translational machinery. Notwithstanding, the presence of DEGs when comparing control and induced conditions signalize the outset of both transcriptional and translational regulation in the initial steps of osteogenesis.

### Osteogenic induction promotes limited gene expression modifications on the first day of induction

**Figure 3:**
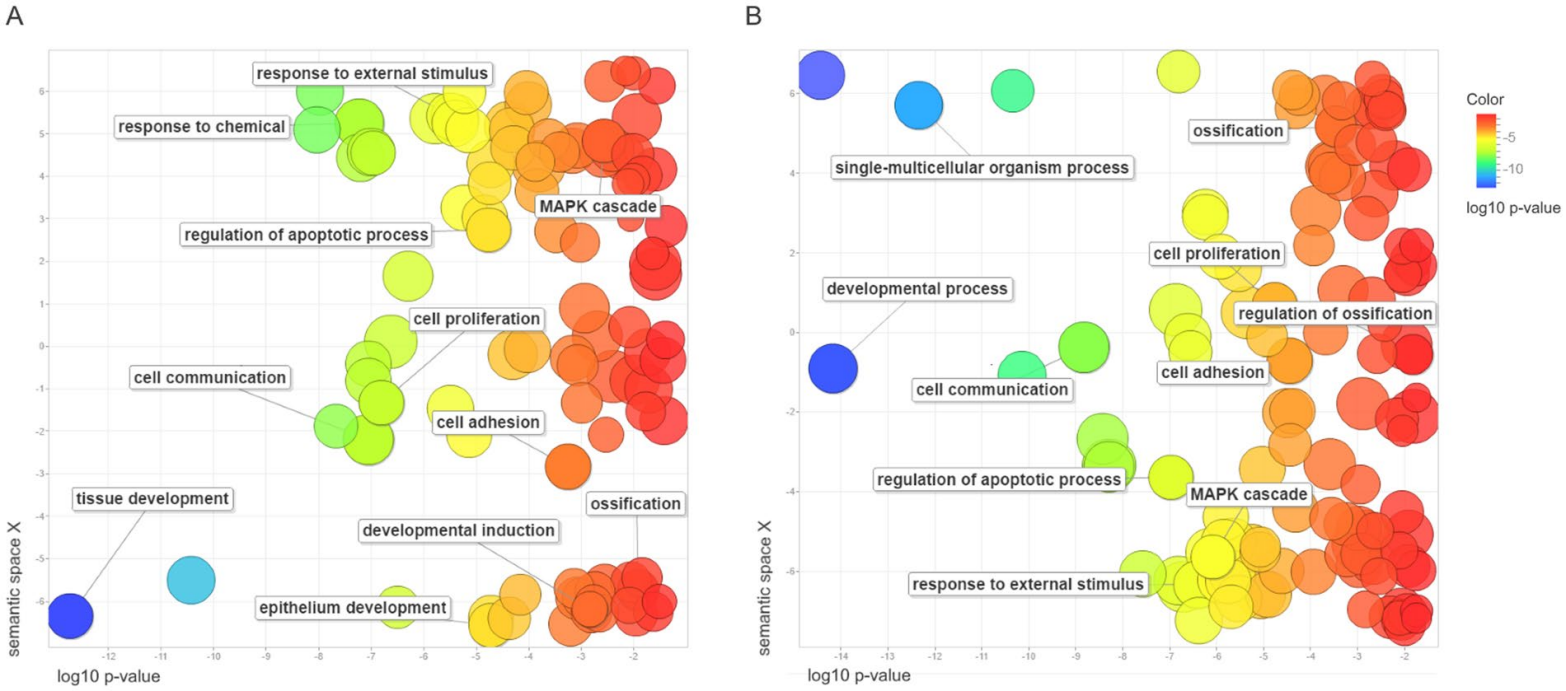
Gene ontology (GO) analysis of biological processes regarding DEGs in polysomal (left chart) and total (right chart) RNA fractions. The figure shows a REVIGO scatterplot of the representative clusters of GO terms obtained with g:Profiler. In the two-dimensional space of the graph, the log10 p-value of each GO after REVIGO analyses is plotted on the x-axis, while the terms are scattered based on their semantic similarities on the y-axis. Bubble color indicates the provided p-value (legend in upper right-hand corner).

Gene ontology (GO) analysis for each set of DEGs from total and polysomal RNA fractions were conducted with g:Profiler (http://biit.cs.ut.ee/gprofiler/). The complete lists of GO analysis concerning cellular components, biological processes, molecular functions and signaling pathways (KEGG) could be found in the Supplementary Tables S4 and S5 (for total and polysomal RNA fractions, respectively). Although we have identified a smaller number of DEGs in the total fraction, the number of GO terms were higher than that found in the polysomal fraction (Supplementary Tables S4 and S5). In order to aid visualization of the GO terms found for biological processes and point out differences between RNA fractions analyzed, we summarized them (removing redundant GOs and grouping them by similarity) using REVIGO (http://revigo.irb.hr/)^15^. Most of the biological processes found were overrepresented in both fractions and are related to cell communication, cell proliferation and adhesion, response to external stimuli and development (Fig. 3). Biological processes such as regulation of cell cycle and cell adhesion have already been reported in other works regarding osteogenesis induction of MSCs, although their analyses have been performed with longer induction-differentiation periods^16,17^.

Based on our previous work showing the strong upregulation of adipogenesis-related genes after three days of differentiation induction^12^, we sought out the first steps of osteogenesis, searching for known markers of osteogenic differentiation. Interestingly, after only 24 h of osteogenic induction, we did not identify key transcriptional regulators of the osteogenesis such as Runt-related transcription factor 2 (*RUNX2*), a transcription factor closely related to osteogenesis that is responsible for activating a set of genes including osterix, osteocalcin, and type 1 collagen, among others^18–20^. Likewise, the study of cell-fate determination during osteogenic and adipogenic differentiation of MSCs made by van de Peppel and colleagues (2017) demonstrated that enriched biological processes and transcription factors that change expression are mostly identical between the lineages within the first four days of induction^9^. Nevertheless, our data on the polysomal and total RNA fractions showed GO clusters related to “ossification” or “regulation of ossification” (Fig. 3A,B) and for “bone mineralization”. In the upregulated gene set, we found important molecules previously related to osteogenesis, such as Bone Morphogenetic Protein 6 (*BMP6*), Forkhead box O1 (*FOXO1*), osteomodulin (*OMD*), Protein Tyrosine Kinase 2 or Focal Adhesion Kinase 1 (*PTK2* or *FAK*), Neuronal Membrane Glycoprotein M6-B (*GPM6B*) and others.

*BMPs* are a class of *TGFβ* family growth factors comprising more than 20 members that act on various biological processes and signaling pathways, primarily through *SMADs* or *MAPKs*. Among its members, *BMP2*, *BMP4*, *BMP6*, *BMP7* and *BMP9* have been described to play an important role in bone formation^21,22^. Regarding *BMP6*, identified in our data, it has been shown that the overexpression of this gene in MSCs induces osteogenic differentiation in a more efficient manner than does *BMP2* overexpression^23^. Furthermore, the increased level of *BMP6* mRNA expression after 24 h of induction suggests that *BMP6* may act as an early regulator for the commitment of the osteogenesis process.

*FOXO1* is another transcription factor related to osteoblast differentiation. Its activity increases in murine MSCs after a few hours of induction with *BMP2*; however, *RUNX2* appears only at later moments^24^, which is compatible with our observed data. However, when *FOXO1* is silenced, the osteogenic markers decrease, as well as calcification^24^. Its influence on osteogenic differentiation was also verified in MC3T3-E1 cells, in which the interaction between *FOXO1* and *RUNX2* were demonstrated^25^. On the other hand, *FOXO1* has also been described as essential for adipogenic differentiation, as knockdown of *FOXO1* in mouse 3T3-L1 preadipocytes showed a decrease in lipid droplet formation when induced to adipocyte differentiation^26^. Thus, the data obtained in the present study and observed elsewhere showed a strong upregulation of the *FOXO1* gene, suggesting that *FOXO1* is an early marker of osteogenesis.

Other molecules, such as GPM6B, also drew our attention due to their relation with the osteogenic process. The absence of *GPM6B* decreased the expression of ALP mRNA and the mineralization capacity, proving to be an important protein in the process of osteogenic differentiation^27^.

Another interesting gene found in our data analysis is PTK2 or FAK. It is a cytoplasmic protein tyrosine kinase present during the development of most tissues. FAK has numerous roles that include influencing cell migration, proliferation, cell-cell and cell-ECM interactions (reviewed by^28^). However, FAK also plays an important role in osteogenic differentiation. Numerous studies have demonstrated that the absence of FAK leads to a decrease in bone mass or bone regeneration *in vivo*^29,30^. In addition, absence of FAK in bone marrow MSCs leads to less proliferation and osteoblast differentiation^30,31^. Another interesting fact is that FAK interacts with the ERK1/2 pathway, which is responsible for the phosphorylation and activation of RUNX2/CBFA-1^31^.

These results suggest that, although we saw a small number of DEGs between culture conditions (CT vs IN) and could not identify key transcriptional factors of the osteogenesis at the beginning of the differentiation (e.g., *RUNX2*), we found a set of genes related to the osteogenic differentiation and that interact with *RUNX2*. As such, it is possible to imagine that an initial molecular signaling begins after 24 h of induction and starts the process of differentiation of hASCs for an osteogenic fate.

### The hASCs need 21 days of osteogenic induction to differentiate

**Figure 4:**
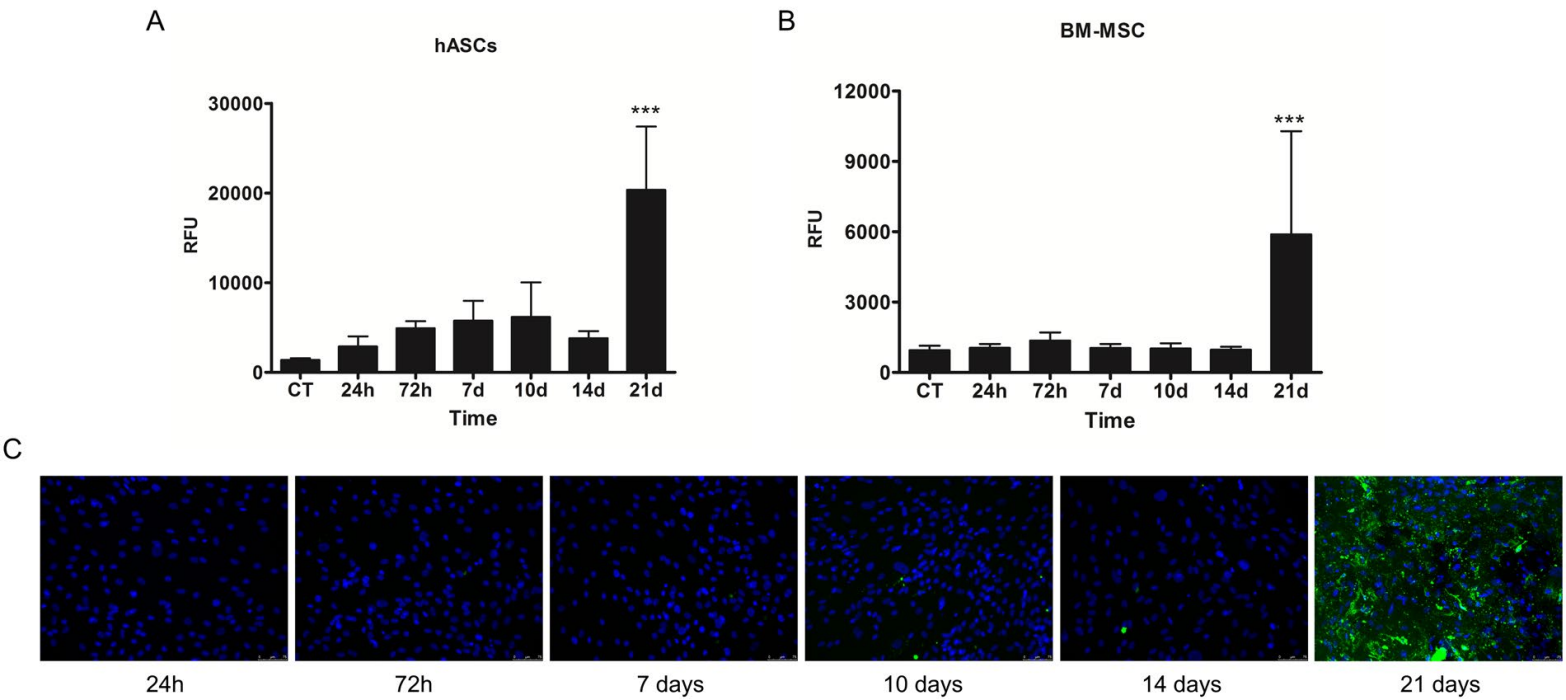
hASCs need 21 days of *in vitro* induction to become osteoblasts. hASCs (**A**) or BM-MSC (**B**) were maintained in differentiation-inducing conditions for different periods of time and allowed to complete osteogenic differentiation (total of 21 days). The graphs represent the means (with standard deviations) of the relative fluorescence units (RFU) for OsteoImage^TM^ staining in each time period. (**C**) Representative images of induced hASCs after 24 h to 21 days of differentiation period. Green fluorescence staining of the hydroxyapatite portion of the mineralized matrix on cell cultures after 21 days. ***denotes p < 0.001.

Considering that key transcriptional factors for osteogenesis were not identified in the first 24 h of osteogenic induction, we tested the induction time required to trigger cell differentiation to the osteogenic phenotype. Classic protocols are based on continuous or alternate induction, where the differentiation occurs in a period of 21 days with the medium being changed every 3–4 days. Thus, we performed a time course for differentiation induction, maintaining induction media in short periods of time (24 and 72 h) and longer periods of time (7, 10, 14 and 21 days). Our results showed that in cell cultures with less than 21 days of induction it was not possible to observe the presence of mineralized compartments, a definitive evidence of the osteogenic process (Fig. 4A,C). Nonetheless, it is important to point out that the evaluation of alkaline phosphatase (ALP) activity and/or expression of type I collagen, osteocalcin, BMP2 and RUNX2 would be early markers of osteogenic differentiation^32^. MAPK/ERK and cAMP/PKA signaling pathway have also been used for investigation of osteogenic induction since they have been implicated in the osteogenic differentiation process, being reported as regulators of proliferation and differentiation of bone cells during osteogenesis^7,33^. Fathi and Farahzadi (2017) provided evidence of osteogenic differentiation showing significant increasing in ALP activity after 16 days of murine ASC cultures treated with zinc sulfate and/or exposed to electromagnetic fields. However, cells treated only with osteogenic induction medium had low levels of ALP during the first days of induction, showing a slight increase at day 20 of the osteogenic differentiation process^8^. Compatible results were found in this study, since the presence of mineralized compartments was observed at the end of osteogenic induction, demonstrating that 21 days of induction were necessary for osteogenic differentiation of hASCs.

Interestingly, hASCs become committed within the first three days of adipogenic differentiation induction^12^, different to osteogenic differentiation. This could be a result of the tissue origin of the adult stem cells, where a cell niche effect could affect the differentiation capacity of the cells. However, when bone marrow stem cells were tested, again, 21 days of continued induction were needed to complete osteogenesis (Fig. 4B). These results indicated that cell commitment to osteogenic differentiation is slower than that for adipogenesis, with gradual changes over the induction time regardless of the origin of MSCs (adipose tissue or bone marrow; Fig. 4A,B).

### IPA analyses reveal osteogenesis related signaling pathways

Complementary analysis of DEGs from the polysomal fraction was conducted with Ingenuity Pathway Analysis (IPA). In addition to GO terms, the IPA displayed cellular growth and proliferation with the highest score of representation, which reached a p-value of 6.67E-18 and 197 related molecules.

IPA showed a series of mRNAs related to signaling pathways among the polysomal DEGs. Among the 5 pathways with the highest p-values, two drew our attention: “Role of Osteoblasts, Osteoclasts and Chondrocytes in Rheumatoid Arthritis” and “p38 MAPK Signaling”. Interestingly, as observed in the diagrams presented using IPA (see Supplementary Fig. S3), the regulated genes in the osteo-related pathway were the ligands, cell receptors or the first intracellular signaling proteins of the cascade; this appears to be consistent with the applied protocol of 24 h of induction. Within this pathway, we found BMPs and activators/inhibitors of the Wnt pathway that were previously described as osteogenic regulators. Our analysis also identified Wnt2 and Wnt11 ligands, that are related to osteogenesis in hMSCs^34^. Furthermore, we have identified other regulated genes related to the Wnt pathway and osteogenesis, such as WNT1-induced Secreted Protein-1 (WISP1) and secreted Frizzled-related proteins (SFRPs or FRZB).

WISP-1 was the first gene discovered as a target for the Wnt pathway; however, further studies demonstrated that this molecule is highly expressed in bone and osteoblasts and influences osteogenesis both *in vivo* and *in vitro* through the interaction with BMP2 or Wnt^35,36^. The FRZB mRNA appeared in our data with a 6.5-fold change increase. The FRZB protein was demonstrated to be capable of modulating the Wnt pathway^37,38^ and improving osteogenic differentiation of MC3T3-E1 cells^37^. In addition, FAK (also regulated according to our experimental data) was able to influence the Wnt pathway during osteogenesis and deletion of FAK decreased Wnt/b-catenin signaling in BMSCs that were induced to undergo osteogenesis^30^.

Additionally, the MAPK signaling cascade was highlighted in our IPA (Supplementary Fig. S3) and g:Profiler analyses. This signaling pathway is vital in the control of cellular behavior by transducing external stimuli. MAPKs are related to osteoblast commitment predominantly through ERK and p38 activities. Interestingly, the GO term “regulation of ERK1 and ERK2 cascade” were also identified and includes some known genes that activate this pathway (Supplementary Tables S4 and S5). The relevant role of ERK1/2 signaling pathway in osteogenic differentiation have been demonstrated previously with different methods of osteogenic induction^7,8,39^. Also, p38 MAPK is another well-known essential pathway in bone formation and differentiation^40,41^. In both cases, kinases are responsible for phosphorylation and activation of genes such as RUNX2 (reviewed by^42^).

### Osteogenic induction modulates hASC adhesion and proliferation

**Figure 5:**
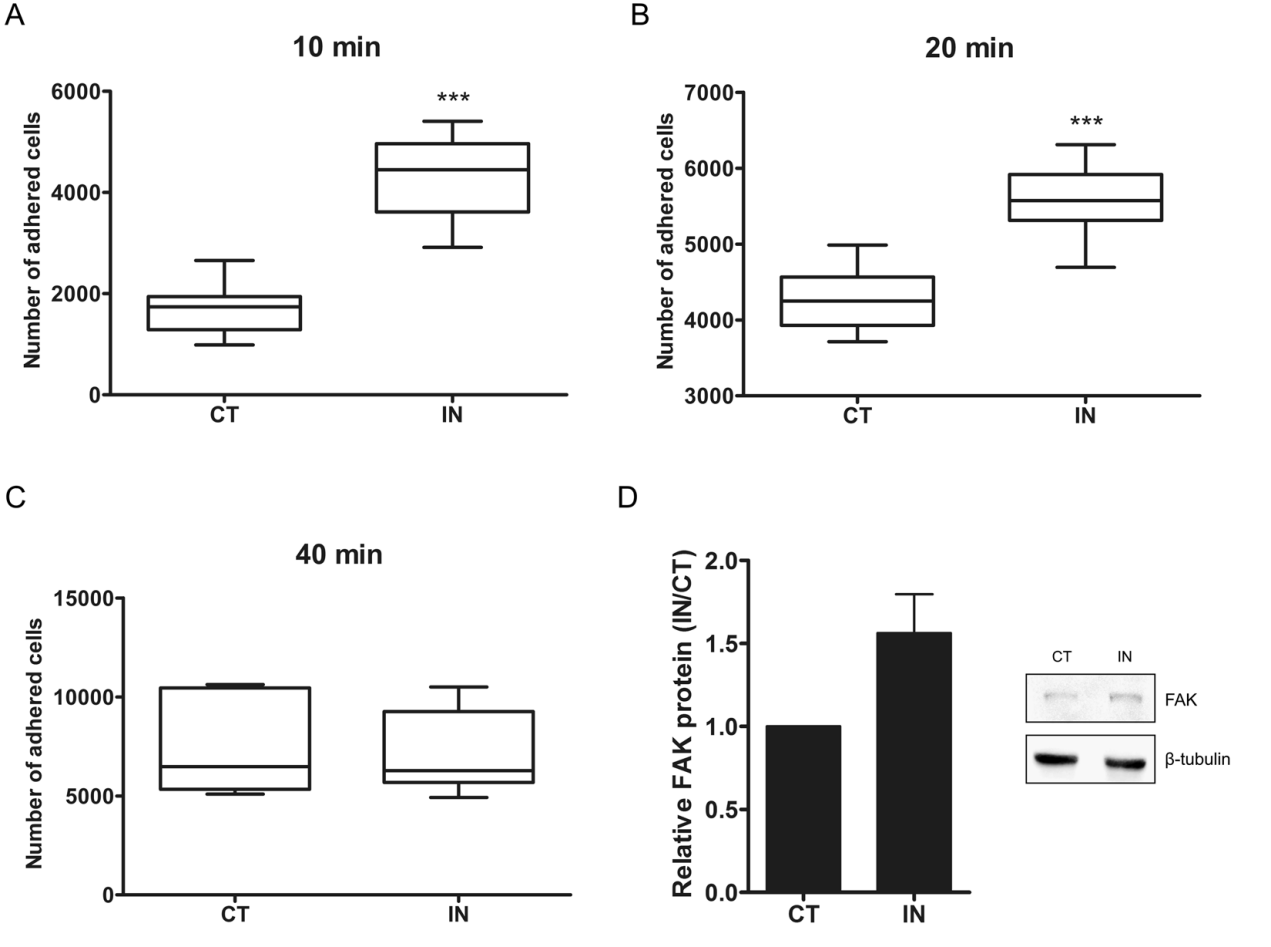
Induced hASCs have improved adhesion capacity. The graphs in A, B and C depict the number of adherent cells obtained by the counting of the nuclei between control and induced conditions. (**A**) Adherent cells after 10 min of culture; (**B**) Adherent cells after 20 min of culture; and (**C**) Adherent cells after 40 min of culture. (**D**) Western Blot analysis and quantification of FAK protein. β-tubulin was used to normalize the amount of FAK under control and induced conditions. Representative images of a cropped Western Blot gel are displayed to evidence the bands, though uncropped images of blots are shown in Supplementary Fig. S4. ^***^denotes p < 0.001.

It is known that mesenchymal stem cells have the ability to adhere to surfaces such as plastics^43^, as well as contain a number of cell surface molecules that promote adhesion^44^. Our GO analysis grouped a series of upregulated genes related to cell adhesion. To understand and confirm these data, we tested the immediate adherence performance of hASCs, evaluating 10, 20 and 40 min after cell seeding. Control and 24 h induced hASCs with osteogenic medium were evaluated. The number of adherent cells was determined by nuclei counting with the support of a high content imaging system. As shown in Fig. 5A–C, induced hASCs were significantly more adhesive (about 2 times more) after the first 10 and 20 min than noninduced cells. This difference was not maintained after 40 min of adhesion, which showed a similar number of adherent cells in control and induced conditions.

As discussed above, FAK protein is related to cell-substrate adhesion and was therefore identified in our analysis among the genes related to adhesion. To confirm the presence of FAK, we performed a western blot assay and compared the amounts of FAK protein between control and induced conditions. After 24 h of osteogenic induction FAK expression was increased in comparison to noninduced cells (Fig. 5D and Supplementary Fig. S4).

Considering the influence of cell shape on MSCs differentiation^45^ and that osteogenic differentiation is favored when the cells are adhered, flattened and spread^46^, our hypothesis is that the increased adhesive capacity of hASCs after 24 h of induction is preparing cells for osteogenic differentiation.

Another GO term that we observed in our results that called our attention was related to cell proliferation. Although proliferation and differentiation are independent cell behaviors, there are a number of studies showing that, in many cases, cells need to reduce or stop proliferation to begin the process of differentiation^47^. As such, we investigated the proliferative profile of the hASCs that were induced to undergo osteogenesis at different time points. The proliferation of induced cells was compared with that of noninduced cells by evaluating nuclei and Ki67 staining. After 10 and 14 days of culture, the induced condition showed significantly higher number of cells than control condition (see Supplementary Fig. S5A–C). The results also indicated that, after 7 and 10 days, induced cells had higher proliferation rates compared to control cells (~10% more cells incorporated Ki67 on the seventh day) (Supplementary Fig. S5D–F). Thus, these results demonstrated that, even under the stimulus of osteogenesis induction, the hASCs continue to proliferate until at least the 14^th^ day of culture.

Investigating the genes described in cell proliferation we found that the “Cell cycle regulator” (RGCC) and “zinc finger and BTB domain containing 16” (ZBTB16) had an increased fold change in their transcript levels in total and polysomal RNA fractions of induced cells. These genes have already been described as being involved in the process of cell proliferation and cell cycle regulation^48–50^. Some studies noticed that during osteogenic differentiation there was an increase in cell number when compared, for example, to adipogenic differentiation^17,46^. Thus, an increase in cell number appears to be a natural feature of the *in vitro* osteogenic differentiation process.

## Conclusion

In summary, the present study showed that, with only 24 h of osteogenesis induction, hASCs were not fully committed to the osteogenic phenotype. The analysis of the polysome-associated transcripts showed that most differentially expressed mRNAs are regulated at this level. Among the upregulated genes, we could not identify canonical pathways or classical transcription factors related to the osteogenic process. Nevertheless, the data revealed other signals and proteins that are related to the initial regulation of osteogenic differentiation, such as *BMP6* and *FOXO1*. An interesting difference between induced and noninduced hASCs was related to cell proliferation and adhesion, in which induced cells displayed an improved capacity compared with control cells. Taken together, our results suggest that a 24 h induction period is not sufficient to trigger and drive *in vitro* osteogenic differentiation of hASCs and that cells need a prolonged stimulus period to be committed to differentiation.

## Material and Methods

### Isolation, characterization, culture and differentiation of hASCs

Human stem cells were obtained from adipose tissue derived from lipoaspirate samples from four female healthy donors aged 27, 32, 44 and 56 years old. Samples were obtained after signed informed consent was provided by the donors or their legal representatives, in accordance with guidelines for research involving human subjects and with the approval of the Ethics Committee of Fundação Oswaldo Cruz, Brazil (approval number 419/07). hASCs were isolated and expanded as previously described elsewhere^51^ with modifications. Briefly, 200 ml of adipose tissue were digested with 1 mg/ml collagenase type I (GIBCO) solution for 30 min at 37 °C, 5% CO_2_ under constant shaking. The cell suspension obtained was centrifuged and treated with hemolysis buffer to remove contaminating erythrocytes. The cells were washed and plated at a density of 1 × 10^5^ cells/cm^2^ in T75 culture flasks in DMEM supplemented with 10% FBS, penicillin (100 units/ml) and streptomycin (100 µg/ml). Flasks were incubated in a humidified incubator at 37 °C with 5% CO_2_. The culture medium was changed every 3–4 days until the cells reached 80% to 90% confluence. All tests were performed with cell cultures at passages 4 to 6. Cell characterization was performed according to the minimal criteria for defining MSCs as established by the International Society for Cellular Therapy^43^. Flow cytometry analysis were conducted as previously described elsewhere^52^ with modifications. At brief, cells were trypsinized and incubated with bovine serum albumin (BSA) 1% diluted in PBS, at 4 °C, for one hour. Then the cells were incubated for another one hour, at 4 °C in the dark, with the following antibodies: FITC-conjugated anti-human CD90, CD34, CD31 and CD19; APC-conjugated anti-human CD73; PE-conjugated anti-human CD45, HLA-DR, CD117 and CD11b. Mouse IgG antibodies (FITC, APC, PE) were used as negative controls. After incubation, the cells were washed for data acquisition at FACSCanto II (Becton Dickinson). At least 10,000 events, for each sample, were collected and analyzed with FlowJo® v.10 software (Flowjo, LLC).

For adipogenic and osteogenic differentiation, hASCs were treated with hMSC Adipogenic Differentiation Medium (hMSC Adipogenic BulletKit, Lonza) or with hMSC Osteogenic Differentiation Medium (hMSC Osteogenic BulletKit, Lonza) respectively, in accordance with the manufacturer’s instructions. Briefly, adipogenic differentiation was induced by cycles of induction/maintenance over 28 days. Induction medium consisted of adipogenic inducers indomethacin, insulin, dexamethasone and IBMX. Osteogenic differentiation was induced with medium containing β-glycerophosphate, ascorbic acid and dexamethasone over 21 days. The medium was replaced every 3–4 days.

Efficiency of adipogenic and osteogenic differentiation was determined by assessing the cytoplasmic accumulation of triglycerides by AdipoRed™ Assay Reagent (Lonza) or by assessing the mineralized extracellular matrix through OsteoImage™ Mineralization Assay (Lonza), respectively.

### Sucrose density gradient separation and RNA purification

Polysomal fractions were prepared with a modified version of the procedure described previously^12^. At brief, hASC cultures at 60 to 70% confluence were induced to osteogenic differentiation (as described in section 2.1) for 24 h, and then the cultures were treated with 0.1 mg/ml cycloheximide (Sigma-Aldrich) for 10 min at 37 °C, followed by trypsinization. The resulting cell pellets were resuspended in 0.1 mg/ml cycloheximide in PBS and was centrifuged (2000 × *g* for 5 min); this step was repeated twice. The cells were lysed by incubation for 10 min on ice with polysome buffer (15 mM Tris-HCl pH 7.4, 1% Triton X-100, 15 mM MgCl_2_, 0.3 M NaCl, 0.1 μg/ml cycloheximide). The cell lysates were centrifuged at 12000 × *g* for 10 min at 4 °C. The supernatants were carefully isolated, loaded onto 10% to 50% sucrose gradients (BioComp model 108 Gradient Master ver. 5.3) and centrifuged at 150000 × *g* (SW40 rotor, HIMAC CP80WX HITACHI) for 160 min at 4 °C. The sucrose gradient was fractionated with the ISCO gradient fractionation system (ISCO Model 160 Gradient Former Foxy Jr. Fraction Collector), connected to a UV detector to monitor absorbance at 275 nm, and the polysome profile was recorded. The total and polysomal RNA fractions were extracted using the Direct-zol^TM^ RNA MiniPrep (Zymo Research) according to the manufacturer’s instructions.

### cDNA library construction and RNA sequencing (RNA-seq)

We used 1 µg of total and polysome-associated RNA fractions for RNA-seq from three independent biological sample replicates. The cDNA libraries were prepared with the TruSeq Stranded Total RNA Sample Preparation kit (Illumina, Inc.), following the manufacturer instructions, and the purified products were evaluated with an Agilent Bioanalyzer (Agilent). The RNA-seq was performed in an Illumina HiSeq platform using RNA-seq kit according to the manufacturer’s recommendation (Illumina, Inc.).

### Data analysis

Mapping and counting was performed with the R package Rsubread^53^ against the new version of the human genome GRCh38. Parameters were set for unique mapping of the reads. For the determination of differential expression between differentiating cells (IN) and undifferentiated stem cells (CT), only those genes with counts of more than 1 per million in at least three conditions were considered. Differentially expressed genes (DEGs) were identified with edgeR Bioconductor package^54^. Correction for multiple testing was performed with FDR (false discovery rate). RPKM values (reads per kilobase per million mapped reads, an expression measure) for each sample were also determined in order to compare the expression of the samples in both conditions. We performed correspondence analysis (COA), a dimension reduction method of the matrix of counts, to explore associations between variables. In COA, it is possible to simultaneously visualize samples and genes, revealing associations between them. Genes, or samples, lying close to each other tend to behave similarly.

Based on initial results of DEGs we used a stringent analysis using a false discovery rate (FDR) threshold of 0.05 and a log2Fold change (logFC). Genes with a logFC > 2 were considered upregulated, and genes with a logFC < −2 were considered downregulated. An enrichment analysis of this set of genes was performed using g:Profiler^55^ (http://biit.cs.ut.ee/gprofiler/) and REVIGO^15^ (http://revigo.irb.hr/) consortium database. Complement analysis was performed with Ingenuity Pathways Analysis (IPA) software, data version 26127183 (Qiagen, Valencia CA, USA).

### Adhesion assay

After induction of osteogenic differentiation for 24 h, cells were trypsinized, plated on 24-well plates (2,5 × 10^4^ cells/well) and kept for 10, 20 and 40 min in a humidified incubator at 37 °C with 5% CO_2_ as previously described^56^. The non-adherent cells were removed by washing twice with PBS. Adherent cells were fixed with 4% paraformaldehyde followed by DAPI staining for counting. The quantitative analysis was performed using an Operetta HTS imaging system (PerkinElmer, Waltham MA, USA) at 100× magnification. Sixty five fields were analyzed and photographed for counting labeled cells with Harmony 3.5.2 software (PerkinElmer).

### Cell proliferation assay

Ki67 and DAPI staining were used to evaluate proliferation of hASCs in the Operetta HTS imaging system. Nine fields of view at 100× magnification were used to perform quantitative analysis in the Harmony 3.5.2 software. Briefly, hASC were seeded at 96-well plates (10^3^ cells/well) and incubated at 37 °C, 5% CO_2_ with DMEM 10% FBS until reached 80% of confluence. Then, osteogenic induction medium was added and maintained in culture for 1, 3, 7, 10 and 14 days. After each period of incubation, cells were fixed with 4% paraformaldehyde, permeabilized with Triton X-100 0,5% (30 min), blocked with BSA 1%, diluted in PBS and stained with Ki67 antibody (Abcam).

### Western blot analysis

Cell extracts were prepared by adding lysis buffer (50 mM Tris-HCl pH 7.5, 150 mM NaCl, 1 mM MgCl_2_, 0.5% Nonidet-40, 1 mM dithiothreitol) supplemented with a protease inhibitor cocktail (Sigma-Aldrich). Cells were removed with a cell scraper, incubated with lysis buffer at 4 °C for 2 h and then centrifuged at 16100 × *g*. Supernatants were collected and treated with SDS sample buffer. Western blot analyses were performed with rabbit anti-FAK (1:1000, Cell Signaling) and rabbit anti-tubulin (1:2000, Cell Signaling) antibodies. The peroxidase-conjugated anti-rabbit IgG was used as secondary antibody. ImageJ software (https://imagej.nih.gov/ij/) was used for the quantitative analysis and different gel images were quantified using linear signal ranges.

### Statistical analysis

Statistical differences were determined by one-way ANOVA followed by Tukey’s test and, for grouped analysis, two-way ANOVA followed by Bonferroni post hoc test. Analyses were performed using GraphPad Prism 5 software. Values of p ≤ 0.05 were considered significant. Data are expressed as the mean ± standard deviation. All experiments were performed with three biological replicates.

### Data availability

The RNA-seq raw data are deposited in the ArrayExpress repository under the number E-MTAB-6298. Analyzed datasets are available in this article and its supplementary files.

## Electronic supplementary material

Supplementary Figures

Supplementary Table S1

Supplementary Table S2

Supplementary Table S3

Supplementary Table S4

Supplementary Table S5
