# Supplementary material for: Gene expression analysis of human adipose tissue-derived stem cells during the initial steps of in vitro osteogenesis
Source: Sci Rep. 2018 Mar 16;8:4739. doi: 10.1038/s41598-018-22991-6 (PMC5856793; doi:10.1038/s41598-018-22991-6)
Supplement: Supplementary file 1 — Supplementary Figures [file 41598_2018_22991_MOESM1_ESM.doc]

**Gene expression analysis of human adipose tissue-derived stem cells during the initial steps of *in vitro* osteogenesis**

Anny Waloski Robert^a,#^, Addeli Bez Batti Angulski^a,#^, Lucia Spangenberg^b^, Patrícia Shigunov^a^, Isabela Tiemy Pereira^a^, Paulo Sergio Loiacono Bettes^c^, Hugo Naya^b^, , Alejandro Correa^a^, Bruno Dallagiovanna^a^, Marco Augusto Stimamiglio^a^,*

**Supplementary figures**


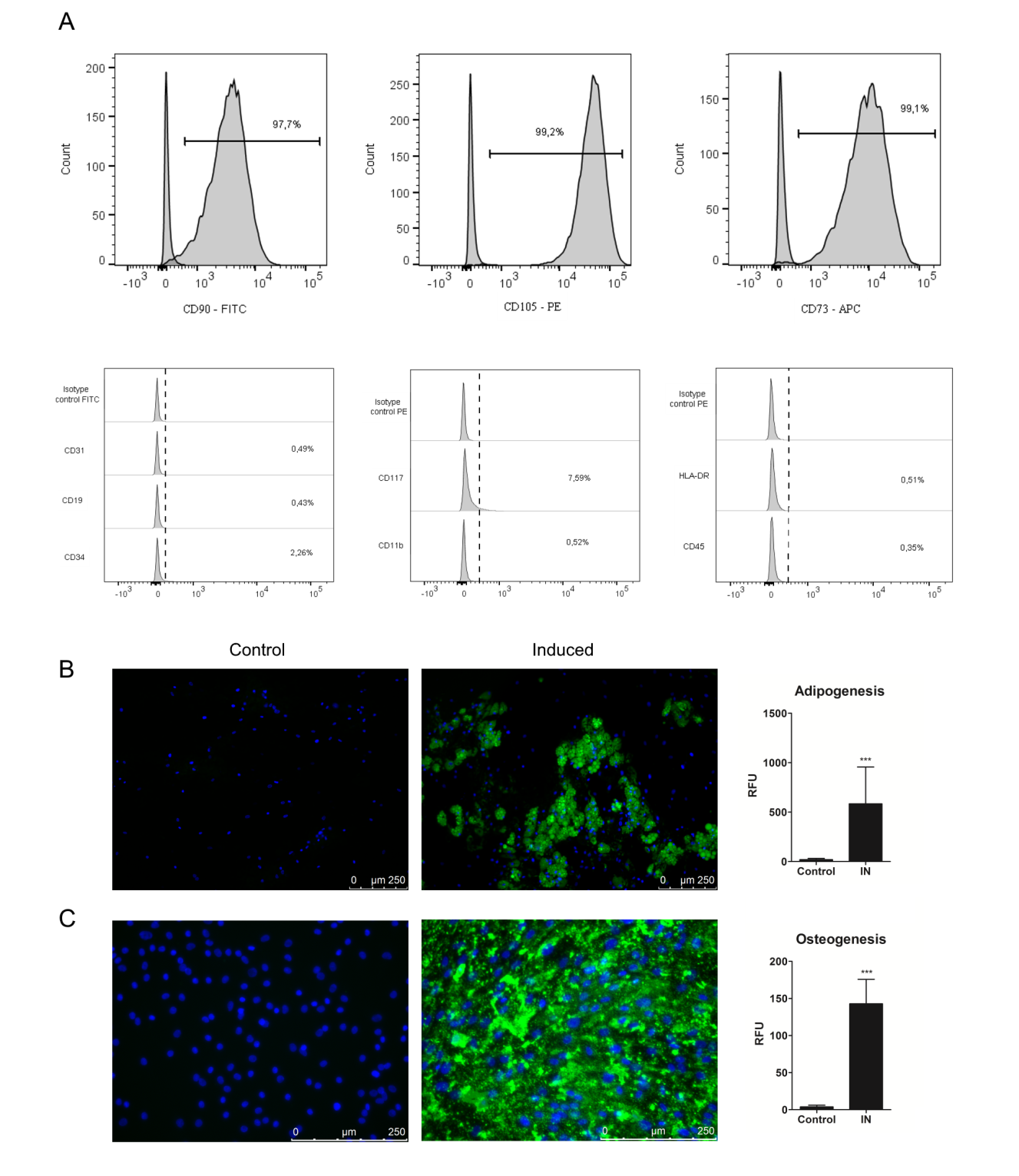


Supplementary Fig. S1: hASCs characterization. A) Flow cytometry histograms for the corresponding cell-surface antigens: positive markers (upper panels; the slim curves on the left represents isotype control fluorescence intensities) and negative markers (lower panels; dashed lines mark isotype control fluorescence intensities for each channel). B and C) Representative images of adipogenic and osteogenic differentiation of hASCs, respectively. The graphs on the right represent the relative fluorescence units (RFU) for AdipoRed^TM^ and OsteoImage^TM^ staining. *** denotes p<0.001.


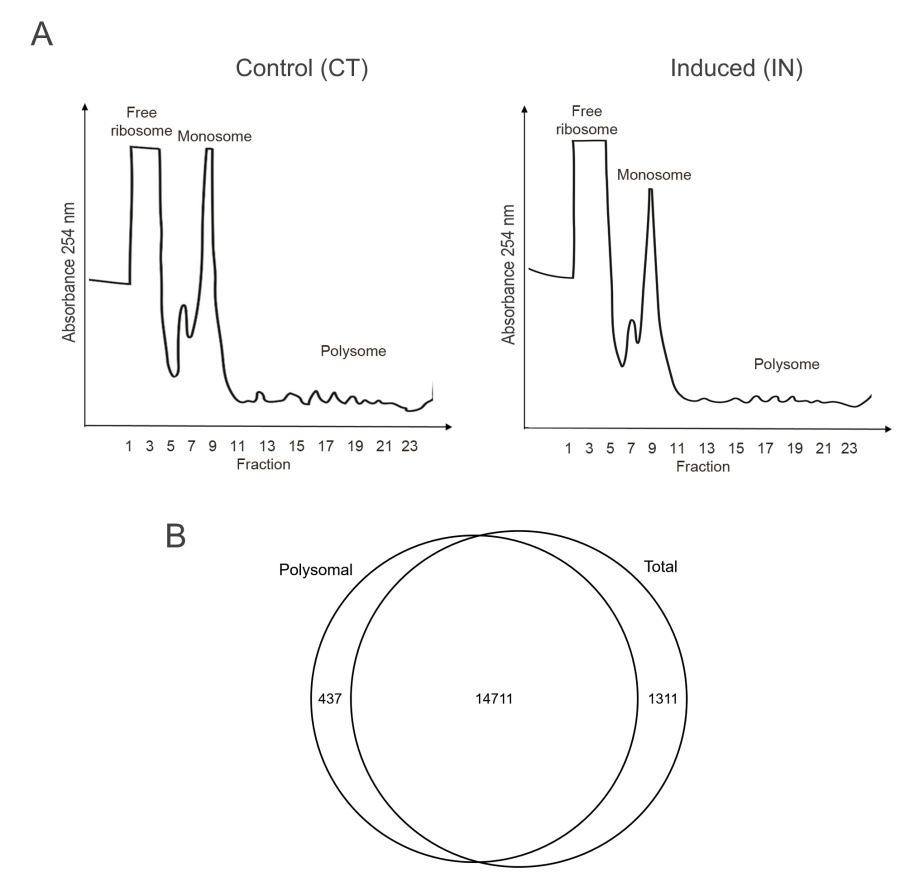


Supplementary Fig. S2: Profiles of polysome and RNA populations on hASCs. A) Polysome profile of 24 h undifferentiated cell cultures (noninduced control, left graph) and 24 h induced cell cultures (induced with osteogenic medium, right graph). B) Venn diagram showing the genes detected in both conditions: polysomal and total RNA populations.


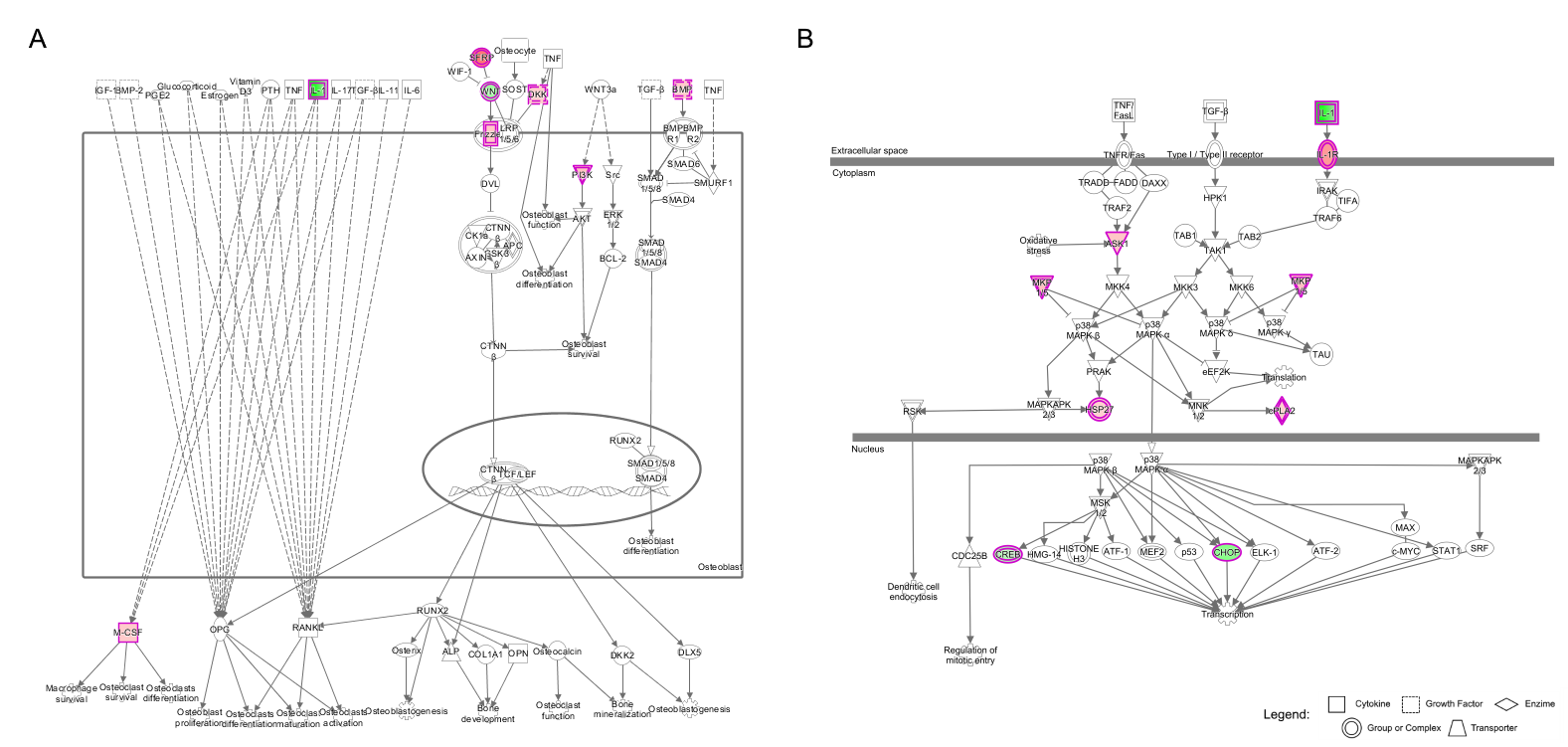


Supplementary Fig. S3: Cell signaling pathways related to osteogenesis as identified by IPA analysis. (A) Role of Osteoblasts, Osteoclasts and Chondrocytes in Rheumatoid Arthritis; (B) p38 MAPK Signaling. Colored boxes emphasize the DEGs found by IPA in the mRNA polysomal fraction in each signaling pathway.


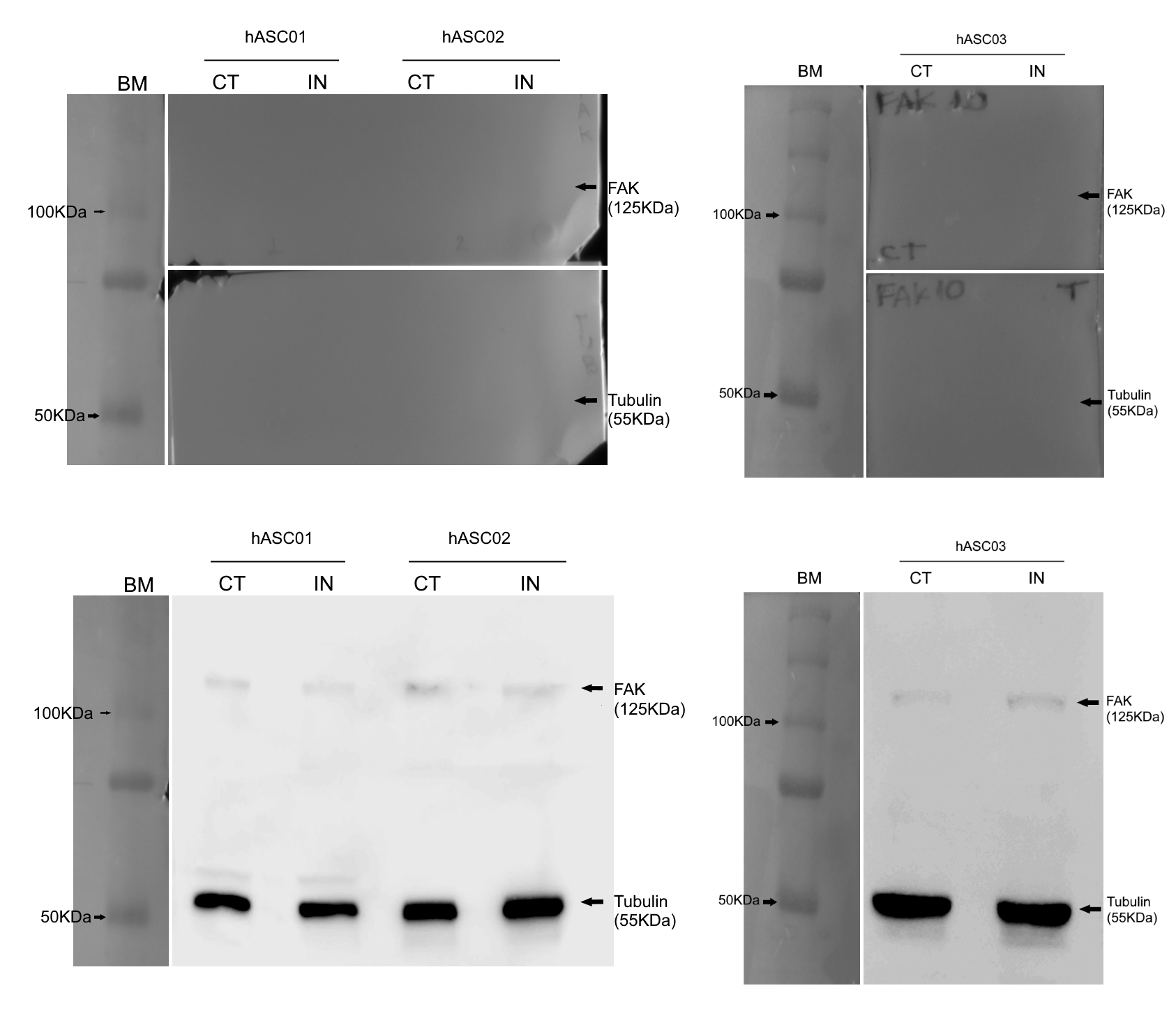


Supplementary Fig. S4: Western Blot (WB) analysis of FAK protein. The images evidence full-length gels assayed with three different samples of hASCs. Protein samples are derived from the same experiment and the blots were processed in parallel. Upper images represent cropped transference membranes with the respective dyed benchmarks. The lower images depict WB performed with anti-FAK and anti-βTubulin antibodies in which benchmark lanes were pasted for reference. BM = Odyssey® (Li-Cor) one-color protein molecular weight marker; CT = control condition; IN = osteogenesis induced condition.


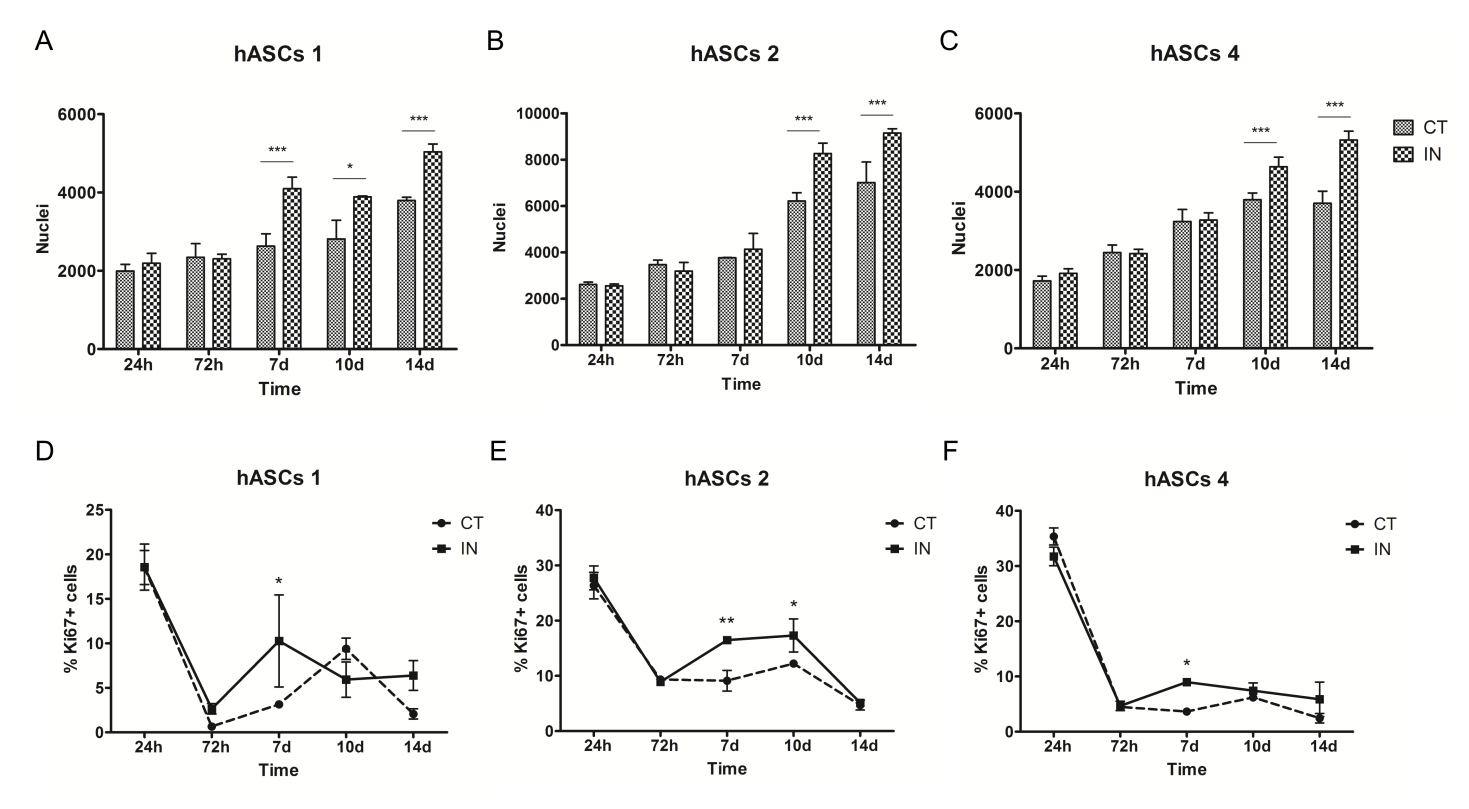


Supplementary Fig. S5: hASCs continue to proliferate during osteogenesis induction. A-C) Graphs depict the total number of cells analyzed at different time points during osteogenic differentiation. D-F) Graphs show the percentage of cells stained for KI67 at different time points during osteogenic differentiation. Data on graphs represent the means (with standard deviations) of stained cells in control and induced conditions. * denotes p<0.05; ** denotes p<0.01; and *** denotes p<0.001.
